# Free Emergency Manual Books Improve Actual Clinical Use During Crisis in China

**DOI:** 10.7759/cureus.4821

**Published:** 2019-06-03

**Authors:** Jeffrey Huang, Peter Hoang, Wayne R Simmons, Jianfeng Zhang

**Affiliations:** 1 Anesthesiology, University of Central Florida College of Medicine, Orlando, USA; 2 Anesthesiology, Hospital Corporation of America West Florida Graduate Medical Education Consortium / Oak Hill Hospital, Brooksville, USA; 3 Anesthesiology, Xiangyang Central Hospital, Xiangyang, CHN

**Keywords:** emergency manuals (em), crisis, clinical use, free book

## Abstract

Introduction

Emergency manuals (EM) are widely implemented and effective tools for anesthesiologists and perioperative teams to manage patients during critical events. Team simulation studies have shown that the use of training aids and checklists decreases human error. Previous research has examined the use of EM at hospitals in the United States, but few studies have explored its impact in an international setting. In this paper, we conduct a nationwide survey in China to assess the implementation and effectiveness of EM in clinical settings.

Methods

Based on the known benefits of using these training aids, we hypothesize that introducing EM will improve team response and reduce errors during crisis management. Copies of the translated Stanford University Operating Room Emergency Handbook were distributed free of charge to hospital anesthesiology departments across China. A survey was then sent out to members of the New Youth Anesthesia Forum, a social networking group of over 100,000 anesthesiologists.

Results

Respondents (n = 818) were separated based on whether or not they received the free EM (yes = 410; no = 408). Our study found that groups who received the manuals demonstrated significantly higher levels of self-review, group study, simulation training participation, and usage during critical events than groups that did not receive the free books (respectively; *p* < 0.001).

Conclusions

These findings strengthen prior evidence suggesting that implementing EM can contribute to the effective management of acute events in a hospital and preoperative setting. Overall, EM can minimize preventable patient risk and benefit anesthesiologists in their clinical practice. These findings indicate that free books can enhance the implementation of emergency manual and actual emergency manual use during critical events.

## Introduction

Physicians, nurses, and staff are carefully trained and expected to perform at a high level of proficiency. Despite this, errors in medicine remain a consequential aspect of patient care. Changing reactions to error and implementing prevention strategies are necessary to reduce consequences in perioperative emergency situations. A few mechanisms have been suggested to combat medical errors including reducing the reliance on memory, improving access to information, and standardizing resources [1]. One such training aid that encompasses these elements are emergency manuals (EM). They have been an effective tool for anesthesiologists and preoperative teams to treat patients efficiently and effectively during acute events [2].

One of the most widely used and concise EM, the Stanford University Operating Room Emergency Handbook, was developed by the Stanford Anesthesia Cognitive Aid Group [2]. They compiled various cognitive aids relevant in a particular health context or scenario into an easily understandable format. Cognitive aids are tools used to help providers recall information that may have already been learned but may be difficult to retrieve in high-stress emergent situations in healthcare and other high-stress industries such as aviation [2-3]. In fact, a simulation-based trial from Harvard found that teams missed 23% of critical events when cognitive aid checklists were not used during OR crises versus 6% when they were used [4]. Prior studies have also showed increased successful emergency usage following participation in critical training simulations by anesthesiology residents. The implications of EM are wide-ranging, but ultimately, they serve to improve physician and staff performance while minimizing any preventable harm to patients.

In the United States, EM are becoming increasingly encouraged and even required in staff training and operating rooms [2,5]. However, their adoption in the international healthcare world remains unclear. To the best of our knowledge, data regarding the EM use in hospital settings in China is still lacking. To this end, free copies of the translated versions of EM were distributed free of charge to hospital anesthesiology departments in China. We conduct multi-institutional surveys in this study to provide data that assess the effectiveness of hospital training for Chinese anesthesiologists and their clinical practice from the free copies of EM. The results also help identify targeted areas for improvement and improve implementation methods for future evaluations.

## Materials and methods

The New Youth Anesthesia forum is the largest anesthesia social network in China. The group comprises over 100,000 registered anesthesiologists and was established on WeChat, a widely used instant messaging service application [6-7]. The New Youth Anesthesia Forum, in conjunction with Zhejiang Xianqin Pharmaceutical Co., Ltd., facilitated the distribution of these free booklets to members in this network. 40,000 copies of the translated Stanford University Operating Room Emergency Handbook have been presented free of charge to hospital anesthesiology departments across China. Only those members who filed a request application received the books. More than 2,000 hospitals applied and received free emergency handbooks.

After receiving permission from Xiangyang Central Hospital, the local hospital authority, we distributed an electronic survey to all members through WeChat via the Wenjuanxing software from March 2018 to April 2018. New messages regarding the questionnaires were released to each member in the network every day. All levels of hospital anesthesiology department providers were encouraged to participate actively. Respondents had the option to use a mobile device or desktop computer to complete the survey and were each supplied a unique IP address to only submit once. Survey questions included basic demographic information, emergency manual usage, and barriers during training and critical events. Answer choices included Yes or No boxes and selection of the best answer from two to five available options. There were no follow up surveys sent out to those who responded. No additional sources of data will be used (i.e. archived records, databases, etc.).

Comparisons between the groups that used EMs during critical events between the two groups were done using the chi-squared (χ²) test. All tests were two-tailed with a type I error rate of 0.05. Mean and standard deviation (SD) were calculated for all quantitative variables.

## Results

The survey was conducted from March 2018 to April 2018. There is a total of 818 responses.

Participants’ demographic data are shown in Table 1. Hospitals in China are classified into three tiers according to the hospital’s ability to provide medical care, medical education, and to conduct medical research. Hospitals are designated as grade I, grade II, or grade III. Physician titles included resident physician, attending physician, and chief physician. The number of years participants have been working are also included in the table. Overall, there is a diverse and representative distribution of physicians participating in this study.

**Table 1:** Demographic data of the participants

Participants data	N	%
Hospital Level		
I	47	5.75
II	420	51.34
III	351	42.91
Title		
Resident physician	232	28.36
Attending physician	361	44.13
Vice-chief physician	172	21.03
Chief physician	53	6.48
Working Years		
Less than 5 years	118	14.43
5-10 years	230	28.12
10-20 years	273	33.37
More than 20 years	197	24.08

The total number of respondents and EM usage of each provider are shown in Table 2.

**Table 2:** Frequency of emergency manuals use in clinical settings EM, emergency manual

Frequency of EM use during a crisis	N	%
0	365	44.62
1	182	22.25
2	145	17.73
3	80	9.78
4	13	1.58
5	2	0.24
>5	31	3.79

Next, barriers to the EM use were assessed using pre-generated responses to the questions shown in Table 3. Participants were able to select multiple responses. Many clinicians cited the fast pace of the operating room as a possible barrier to the use of EM. In addition, there appeared to be a lack of adequate training for critical events (73.59%, *n* = 602) and more specifically simulation training (66.87%, n = 547). Thus, an area of need would be to address the education barrier of using EM by implementing more instructors and training sessions.

**Table 3:** Barriers to emergency manuals use during training and critical events EM, emergency manuals

Survey questions about barriers of EM use	N	%
What is the biggest obstacle to using the EM?		
Events in the operating room happen too quickly	456	55.75
Insufficient staff to help	397	48.53
Too nervous to use	276	33.74
Why did you not use the EM during the crisis?		
Lack of sufficient training programs	602	73.59
Doctors must remember these protocols	283	34.60
Do not know how to use	139	16.99
My colleagues may not approve	98	11.98
Why did you not participate in simulation training?		
No one organized simulation training	547	66.87
No teacher	277	33.86
Clinical work too busy	246	30.07

Another important aspect of study was whether EMs improved confidence, organization, and cooperation during actual critical events. In our study, we are interested in whether EM use can increase one’s confidence, which may reduce errors during crisis. In Table 4, participants were asked about these specific components. Interestingly, the majority of responses indicated that EM does improve one’s confidence (86.55%, *n* = 708), team organization (80.81%, *n* = 661), and team cooperation (82.89%, *n* = 678) during real emergency situations.

**Table 4:** Participant response to value of emergency manuals EM, emergency manuals

Survey questions of value of EM	N	%
EMs improve confidence to manage crisis		
Yes	708	86.55
No	110	13.45
EMs made crisis management more organized		
Yes	661	80.81
No	157	19.19
EMs improve OR crisis management team cooperation		
Yes	678	82.89
No	140	17.11

Respondents (*n* = 818) were then separated based on whether they received or did not receive the free EM (yes = 410, no = 408). As shown in Table 5, our study found that groups who received manuals demonstrated significantly higher levels of self-review, group study, simulation training participation, and usage during critical events than groups that did not receive the free books (respectively; *p* < 0.001). Overall, EMs do benefit the operative and post-operative environment by improving confidence and efficiency in critical situations.

**Table 5:** Frequency of self-review, group study, simulation participation, and EM use during critical events EM, emergency manuals

	Applied and received free books		Did not apply and receive free books		P value
	Yes	No	Yes	No	
Self-review	397	13	223	185	<0.0001
Group study	310	100	120	288	<0.0001
Simulation	196	214	100	308	<0.0001
Actual use	289	121	137	271	<0.0001

## Discussion

Subjective findings of this study found in Tables 2 and 5 demonstrate a need for familiarization, self-review, but also training on how to use the manual which correlates with previous studies [8-9]. An article regarding previous large-scale implementation in China focused on different forms of simulation training performed in China and included interactive lectures regarding why, how, and when to use emergency manuals which were included in an overall implementation movement with multiple forms of simulation including lecture, workshop, demonstrations and simulation competition [10]. More must be done than merely book placement as has been shown with a past attempt to integrate in the US [11]. Successful integration strategies throughout the literature emphasize that leadership engagement in simulation and training is more likely to be successful which was demonstrated previously in China as well [12-13]. A “Train a trainer” approach to emergency manual simulation integration yielded high levels of simulation potentiation stemming from leadership commitment to organize training at their own facilities with as high as 40% fulfillment after only two months [14].

Barriers to emergency manual use were addressed in Table 3 in our survey of which the greatest factor was lack of training, particularly simulation training as attested to by over 66% of participants. The previously mentioned study in China one year after large scale multi-institutional implementation showed strong correlation of EM usage to simulation training with roughly 69% of surveyed respondents having participated in multidisciplinary simulation training with 70% reporting EM use during at least one critical event in the previous six months, proving a largely successful implementation plan as a whole [6]. Another study out of China demonstrated that there are multiple forms of emergency manual training that can be utilized such as a simulation competition which was used, namely “Simulation Wars” to promote emergency manual use which yielded significantly increased utilization during critical events of roughly 85% after one year following the competition that contributed directly to participants’ willingness or ability to use emergency manuals [15].

Another barrier that was considered a significant factor was the fast pace in the OR attested to by 55% of the participants. However, it is difficult to assess a direct correlation to OR pace as opposed to unfamiliarity with the manual or lack of training being attributed to the OR pace as a confounding variable. Multiple studies from the field of aviation have shown that when an emergency manual is combined with recurrent training, it can be used effectively in such high paced critical events [8-11]. The survey from our study correlates with these aviation articles in that groups within the study at hand who utilized the emergency manual as a resource were also the same participants which demonstrated significantly higher levels of self-review, group study and simulation training.

Finally, possibly the most influential finding from our survey and analysis shows >80% of our diverse participants agreed in Table 4 that EMs improve confidence in team organization and team cooperation during real emergency situations. These findings correlate with and strengthen prior evidence suggesting that implementing emergency manuals in a perioperative setting can contribute to the effective management of acute crises [16-18]. Added significant benefit from our study is the comprehensive and diverse population with a variety of represented levels of education and professional experience who have used emergency manuals in real life situations as opposed to only simulation as seen in Table 1 which demonstrates a universal applicability in the field of Anesthesia as has been found in previous studies [19]. With adequate training, both regarding the outline and contents of the manual itself and additional simulation using an emergency manual, coupled with enthusiasm and participation from local leadership a plan for integration and real-world use in perioperative crises can be successful in benefiting anesthesiologists in their clinical practice by minimizing preventable patient risk.

A limitation from this study is pre-formed survey responses from participants, which does not allow for subjectivity or in-depth vision into cause-and-effect such as in the discussion of barriers to emergency manual utilization. Questions and possible responses in the survey were kept concise to maximize the number of participants, but a drawback could be an inability to gain a deeper understanding into the participant’s response. While it is not the focus of this study, future studies could include qualitative survey data to support and strengthen quantitative findings. This would allow more subjective insight into the use and implementation of emergency manuals. It would also be useful to better define operational terms. For instance, anesthesiology training in China lacks standardization, so there may be varying perceptions on what constitutes a critical event among healthcare providers.

## Conclusions

These findings strengthen prior evidence suggesting that implementing EM can contribute to the effective management of acute events in a hospital and preoperative setting. EM can minimize preventable patient risk and benefit anesthesiologists in their clinical practice. This study indicates that free books can enhance the implementation of EM and actual EM use during critical events.

## Appendices

Questionnaire

1.      What is your hospital level?

a.      I

b.     II

c.     III

2.     What is your title?

a.      Resident physician

b.      Attending physician

c.      Vice-chief physician

d.      Chief physician

3.      How many years of working experience do you have?

a.      Less than 5 years

b.      5-10 years

c.      10-20 years

d.      More than 20 years

4.      Did you receive a copy of the free emergency manual?

a.      Yes

b.      No

5.      Did you have a digital copy of the emergency manual on your smart phone?

a.      Yes

b.      No

6.      In the past year, did you self-review the emergency manual?

a.      Yes

b.      No

7.      Did your department organize group study of the emergency manual?

a.      Yes

b.      No

8.      In the past year, did you use the emergency manual during actual crisis?

a.      Yes

b.      No

9.      How many times did you use the emergency manual during critical events?

a.      0

b.      1

c.      2

d.      3

e.      4

f.       5

g.      >5

10.  What is the biggest obstacle to using the emergency manual?

a.     Events in the operating room happen too quickly

b.     Insufficient staff to help

c.     Too nervous to use

11.  Why did you not use the emergency manual during the crisis?

a.      Lack of sufficient training programs

b.      Doctors must remember these protocols

c.      Do not know how to use

d.      My colleagues may not approve

12.  Why did you not participate in simulation training?

a.      No one organized simulation training

b.      No teacher

c.      Clinical work too busy

13.  Did emergency manuals improve your confidence to manage crisis?

a.      Yes

b.      No

14.  Did emergency manuals make crisis management more organized?

a.      Yes

b.      No

15.  Did emergency manuals improve team cooperation in the operating room during crisis management?

a.      Yes

b.      No

## References

[1] Leape LL (1994). Error in medicine. JAMA.

[2] Goldhaber-Fiebert SN, Howard SK (2013). Implementing emergency manuals: can cognitive aids help translate best practices for patient care during acute events?. Anesth Analg.

[3] Hepner DL, Arriaga AF, Cooper JB (2017). Operating room crisis checklists and emergency manuals. Anesthesiology.

[4] Arriaga AF, Bader AM, Wong JM (2013). Simulation-based trial of surgical-crisis checklists. N Engl J Med.

[5] Goldhaber-Fiebert SN, Lei V, Nandagopal K, Bereknyei S (2015). Emergency manual implementation: can brief simulation-based or staff trainings increase familiarity and planned clinical use?. Jt Comm J Qual Patient Saf.

[6] Huang J, Wu J, Dai C (2018). Use of emergency manuals during actual critical events in China: A multi-institutional study. Simul Healthc.

[7] Huang J, Gao H (2016). Regional anesthesia practice in China: a survey. J Clin Anesth.

[8] Watkins SC, Anders S, Clebone A (2016). Mode of information delivery does not effect anesthesia trainee performance during simulated perioperative pediatric critical events: a trial of paper versus electronic cognitive aids. Simul Healthc.

[9] Watkins SC, Anders S, Clebone A (2016). Paper or plastic? Simulation based evaluation of two versions of a cognitive aid for managing pediatric peri-operative critical events by anesthesia trainees: evaluation of the society for pediatric anesthesia emergency checklist. J Clin Monit Comput.

[10] Huang J (2017). New ways explored to promote emergency manual simulation training in China. APSF Newsletter. https://www.apsf.org/article/new-ways-explored-to-promote-emergency-manual-simulation-training-in-china/.

[11] Neily J, DeRosier JM, Mills PD (2007). Awareness and use of a cognitive aid for anesthesiology. Jt Comm J Qual Patient Saf.

[12] Goldhaber-Fiebert SN, Macrae C (2018). Emergency manuals: how quality improvement and implementation science can enable better perioperative management during crises. Anesthesiol Clin.

[13] Banguti PR, Mvukiyehe JP, Durieux ME (2018). The World Health Organization surgical safety checklist: Happy 10th birthday!. Anesth Analg.

[14] Huang J (2018). Successful implementation of a two-hour emergency manual (EM) simulation instructor training course for anesthesia professionals in China. APSF Newsletter. APSF.

[15] Huang J, Parus A, Wu J, Zhang C (2018). Simulation competition enhances emergency manual uses during actual critical events. Cureus.

[16] Fowler AJ, Agha RA (2013). In response: simulation-based trial of surgical-crisis checklists. Ann Med Surg (Lond).

[17] Goldhaber-Fiebert SN, Pollock J, Howard SK (2016). Emergency manual uses during actual critical events and changes in safety culture from the perspective of anesthesia residents: a pilot study. Anesth Analg.

[18] Krombach JW, Marks JD, Dubowitz G (2015). Development and implementation of checklists for routine anesthesia care: a proposal for improving patient safety. Anesth Analg.

[19] Marshall S (2013). The use of cognitive aids during emergencies in anesthesia: a review of the literature. Anesth Analg.

